# Algorithmic seduction: ethical boundaries in AI-powered consumer nudging

**DOI:** 10.3389/fpsyg.2026.1675412

**Published:** 2026-03-16

**Authors:** Shamini James, Karthik S., Binu Thomas, Josheena Jose

**Affiliations:** 1 Kalasalingam Academy of Research and Education (Deemed to be University), Krishnan Kovil, India; 2 Amal Jyothi College of Engineering, Kottayam, India; 3 Marian College Kuttikkanam Autonomous, Kuttikkanam, India; 4 Christ College Autonomous Irinjalakuda, Irinjalakuda, Kerala, India

**Keywords:** AI nudging, algorithmic persuasion, decision neuroscience, digital autonomy, neuro ethics, predictive personalization

## Introduction

In today's age of artificial intelligence, the process of persuasion has shifted from human creativity to machine-based programming. Instead of using smart wording or attractive visuals, modern advertising now depends on machine learning algorithms that create customized content by analyzing users' real-time actions (Matz et al., 2017). These AI-powered methods work on a much wider and more subtle level than traditional advertising, which brings up serious ethical questions about how people are being influenced (Kumar and Suthar, 2024). Unlike earlier forms of marketing, this new type happens within complicated and often hidden digital systems, where psychological techniques are built into the code—usually without users fully realizing it (Collins and Moons, 2019).

This paper explores how AI-based marketing makes it difficult to clearly separate genuine choice from subtle pressure. It suggests that the ethical side of algorithmic influence is still not well studied in psychology. These systems do more than assist decision-making—they shape preferences, take advantage of emotional or mental weaknesses, and challenge the basic idea of personal freedom (Shabbir et al., 2020). A new perspective is required—one that treats algorithmic persuasion as both a psychological and ethical matter, not just a technical or business issue. This way of thinking matches recent psychological studies that examine the impact of new technologies on society (Volti and Croissant, 2024).

In this paper, the term “algorithmic seduction” does not refer to emotional or romantic attachment to machines. Rather, it describes a form of subtle, data-driven influence that operates largely below conscious awareness by shaping attention, preferences, and habits through personalized timing, design, and feedback loops. It denotes a mode of influence that is less about rational argument and more about the strategic orchestration of digital environments to steer behavior.

## Redefining persuasion in the algorithmic age

Conventional methods of persuasion usually involve a clear purpose, structured message delivery, and a recognizable speaker or source. But in today's world of algorithms, persuasion has changed into something more like predicting and shaping people's behavior (Wekalao et al., 2025). AI systems now create personalized content and user experiences by using real-time data about how people act online (Vallabhaneni et al., 2024). This makes persuasion an ongoing and flexible process. These systems also use common psychological tricks—like fear of missing out, avoiding loss, and following what others do—to gently push users toward certain results, usually for business purposes (Phan and Hoai, 2025).

Unlike persuasion from a human, which gives people a chance to think and decide, algorithm-based nudging often skips this thinking process. The persuasive signals are hidden inside complex digital systems, so users cannot easily tell when or how they are being influenced (Brink et al., 2024). The one doing the persuasion is no longer a person, but an intelligent system designed to grab attention and keep people engaged (Kleinberg et al., 2024).

This change creates an imbalance in power and knowledge, which weakens the user's freedom to choose. The system is hidden, the one influencing is unknown, and the final goal is already decided (Santoni de Sio et al., 2024). To understand persuasion in this new situation, we need to combine ideas from psychology with ethical thinking. It is not enough to ask if the influence is effective—we also need to ask if it respects people's freedom, protects their ability to think clearly, and allows them to give proper consent (Duminicǎ and Ilie, 2024).

## Digital impulsivity and the engineering of desire

Consumer psychology and neuroeconomics have studied impulsive buying for a long time as a mental and emotional reaction to things in the environment (Verplanken and Sato, 2011). These things can be special time-limited offers, eye-catching visuals, or emotional content that make people act quickly without thinking too much. Artificial intelligence makes this even stronger by finding, personalizing, and showing these cues at the perfect time and in the right situation (Vishnu and Raheem, 2013). Data about user behavior—like how fast they scroll, what they click, what they bought before, or even body signals—is always being collected to train smart systems that can guess when a person is likely to be influenced.

This creates a repeating cycle: the more data AI systems collect, the better they can predict what users will do; the better the predictions, the more powerful the suggestions become; and the more successful these suggestions are, the more new data is collected (Collins and Moons, 2019). In this loop, the user's freedom slowly becomes weaker because their actions are shaped more and more by the system. The user is not just reacting to normal advertisements anymore—they are moving through a personalized system full of predictions and hidden triggers (Asad et al., 2021; Calo, 2013).

Over time, these small and controlled interactions can become habits, making it hard to see the short-term weaknesses like boredom, tiredness, or stress—not to help the user, but to use them (Chintala, 2024). This change from simple persuasion to addictive behavior needs serious ethical attention and more psychological studies to understand how this kind of digital design affects the human mind (Flayelle et al., 2023).

## From persuasion to predation: ethical gray zones

Persuasion and force are part of the same moral scale, and the difference between them often depends on things like intention, honesty, and whether the user knows what is happening. For example, a message reminding a user about a saved item might be seen as okay. But more aggressive methods—like using mood-detecting systems to sell expensive products to people who are feeling emotionally weak—take persuasion to a harmful level (Calo, 2013). In these situations, it's not just about helping the user choose, but about using their temporary weakness for profit.

This creates serious concerns in the field of neuroethics. As persuasive technologies start to affect people's emotions and thinking, the idea of psychological freedom becomes very important. Ienca and Andorno (Ienca and Andorno, 2017) talked about something called “mental privacy”—which means people have a right to keep their thoughts safe and free from outside influence without permission. When AI systems secretly change a person's choices by guessing their feelings or behavior, they not only break this mental privacy but also weaken the meaning of giving true consent.

Because of these changes, psychology needs to take another look at where to draw the line between good persuasion and harmful manipulation. The line is crossed when people are influenced without knowing it, especially in situations where they are more weak or sensitive, and when the goal is not for their benefit (Sabour et al., 2025). This unclear area needs stronger ethical attention and proper rules to guide how AI systems are made and used.

## The illusion of consent and the myth of rational choice

Supporters of algorithm-based nudging often say that users give permission for these systems by agreeing to terms and conditions. They believe this means users accept data-based personalisation. But in truth, digital consent is much more complicated (Zhang and Sundar, 2019). The consent is usually hidden inside long, difficult documents that most people do not read or fully understand. This weakens the idea of making informed choices and turns consent into a formality instead of a real decision (Toch et al., 2012).

Also, psychology studies often question the idea that consumers always think and act in a fully logical way. Mental limits like bounded thinking, emotional bias, and decision tiredness affect our ability to make clear and careful choices (Yarosh et al., 2021). In online spaces that are built to keep people engaged, users face too much information and too many choices, which makes it even more difficult to stay in control of their own decisions (Chen et al., 2009).

Algorithm-based nudges work in a way that people often do not notice. They change behavior using small and hidden clues in how a website or app looks or how the content is shown. These small actions are hard to see and almost impossible to trace back later, which makes it harder to know if the person acted on their own or was guided (Khan, 2021). When users don't know they are being influenced and cannot really choose to say no, their consent becomes unclear both ethically and psychologically.

## AI, behavioral prediction, and the commodification of vulnerability

One of the most worrying things about algorithmic persuasion is that it can turn a person's mental weakness into something that makes money. Today's AI systems don't just group people by age or interests—they study emotions and behaviors to find the times when someone is more likely to be influenced. This method, called predictive psychographics, lets platforms target people based on short-term feelings like loneliness, stress, or tiredness.

For example, someone who can't sleep at night might start seeing ads for addictive online content or expensive health products at the time when they are most weak. These systems don't wait for the person to say what they need—they guess it in advance by watching how the person behaves and uses their device (Khan, 2021). What looks like a helpful or personalized service is actually a planned way to take advantage of someone's temporary weakness.

Turning human weakness into a way to make money brings serious ethical problems. It uses normal changes in mood, focus, and thinking as chances to influence people for business reasons (Hassan et al., 2022). These actions usually happen without the person knowing, without clear permission, and without any way to say no (Pagoto, 2025). Because of this, they go against new ideas about protecting the mind and respecting people's dignity online.

Psychological science needs to quickly deal with these unfair practices. This means speaking up for better rules to stop emotional profiling and supporting systems that help users without using their pain or problems to make profit.

## Toward a framework for ethical AI-persuasion

The ethical concerns raised in this paper are not primarily technological but psychological. The central problem is not that AI systems personalize content, but that algorithmic persuasion increasingly interferes with the psychological conditions required for autonomous choice. Existing regulatory frameworks focus on legality, transparency, or data protection, yet they do not adequately address how influence operates at the level of attention, self-regulation, and situational vulnerability. To respond to this gap, this paper proposes a psychology-informed ethical framework for evaluating AI-based persuasion.

The framework is explicitly derived from the three central abuses identified earlier in the paper: (1) opacity and hidden manipulation, (2) the illusion of consent, and (3) the commodification of psychological vulnerability. Each of these abuses corresponds to a distinct psychological risk that undermines autonomy in practice. Opacity impairs awareness of influence; illusory consent weakens the capacity to resist or revise decisions; and vulnerability commodification exploits transient cognitive and emotional states that compromise self-regulation. Ethical evaluation must therefore focus not on formal compliance alone, but on whether persuasive systems preserve the psychological capacities necessary for autonomous decision-making.

From this analysis, autonomy is understood not as a binary property secured by consent, but as a set of psychological conditions that must be maintained at the moment of choice. These conditions include awareness of influence, the ability to contest or reverse decisions, proportional exposure to persuasive cues, protection during vulnerable states, and the long-term preservation of cognitive integrity. The five ethical principles proposed below are derived directly from these conditions. They are neither arbitrary nor exhaustive; rather, they form a coherent and non-redundant set addressing distinct psychological risks.

The principles are ordered according to a psychological logic, progressing from the minimal conditions of awareness to the preservation of long-term autonomy.

First, Noticeability of Influence. Autonomous choice presupposes that individuals can recognize when influence is occurring. When persuasive cues operate below conscious awareness—embedded in interface design, ranking systems, or adaptive feedback loops—they bypass reflective judgment. Noticeability therefore requires more than formal disclosure; it demands that algorithmic steering be sufficiently salient for users to understand that their behavior is being shaped. This principle directly responds to the problem of opacity and hidden manipulation by restoring attentional awareness as a precondition of autonomy.

Second, Contestability and Reversibility. Awareness alone is insufficient if users lack meaningful opportunities to resist, modify, or undo algorithmic influence. Psychological autonomy includes the capacity to revise preferences, counter impulsive responses, and reverse decisions made under pressure. Ethical AI-persuasion must therefore allow users to contest personalization, adjust targeting mechanisms, and reverse outcomes such as purchases or subscriptions. This principle addresses the illusion of consent by shifting emphasis from formal agreement to practical control.

Third, Proportionality of Personalization. Persuasive intensity should be limited to what is necessary to support user goals. Psychological research on impulsivity and cue-reactivity demonstrates that excessive urgency framing, repetition, or hyper-personalized triggers can overwhelm deliberative processes and produce compulsive behavior. When personalization is optimized solely for engagement or conversion, it risks becoming coercive in effect even if legally permissible. Proportionality functions as a safeguard against escalation, ensuring that persuasion does not exceed the user's capacity for reflective choice.

Fourth, Vulnerability-Sensitive Protection. A defining risk of algorithmic persuasion is its ability to infer and exploit transient psychological states such as fatigue, stress, loneliness, or emotional distress. These situational vulnerabilities heighten susceptibility to influence and weaken self-regulation. Ethical persuasion requires that such states be protected rather than monetized. This principle responds directly to the commodification of vulnerability by demanding adaptive restraint—reducing persuasive pressure during vulnerable moments instead of intensifying it.

Fifth, Cognitive Integrity and Digital Wellbeing. Beyond individual decisions, algorithmic persuasion can gradually reshape habits, attention patterns, and self-regulatory capacity. Ethical evaluation must therefore consider long-term effects on cognitive integrity, including sustained attention, impulse control, and reflective agency. Digital wellbeing is defined here not as user satisfaction or engagement, but as the preservation of these psychological capacities over time. Safeguards such as cooling-off periods, reduced exposure, and reflective prompts exemplify measures that protect cognitive integrity in practice.

Taken together, these principles constitute a normative evaluative framework, not a technical design checklist. They provide psychology-based criteria for assessing whether AI-driven persuasion preserves or undermines autonomy in practice. While existing regulations address important baseline risks, they do not capture the lived psychological impact of influence at the moment of decision. This framework is therefore intended as a complementary ethical layer, grounded in psychological science, for evaluating persuasive systems beyond formal compliance.

## Reclaiming autonomy in the age of intelligent persuasion

Autonomy in algorithmically curated environments cannot be reduced to awareness or consent alone. As persuasive systems increasingly shape attention, timing, and choice architecture, autonomy must be understood as a psychological capacity that requires active support. This section therefore does not reiterate specific abuses or ethical prescriptions, but reflects on the broader implications of intelligent persuasion for psychological agency.

In traditional ethical accounts, autonomy is often treated as a stable attribute secured through informed consent. However, psychological research shows that autonomy is fragile, context-dependent, and sensitive to cognitive load, emotional states, and environmental cues (Thirumalai and Sinha, 2013). Algorithmic persuasion exploits precisely these conditions by operating continuously, adaptively, and often invisibly. As a result, individuals may appear to choose freely while their attentional focus, impulse control, and evaluative processes are systematically shaped by external systems (Bashir et al., 2015).

Reclaiming autonomy under these conditions requires a shift in ethical emphasis—from protecting abstract rights to sustaining concrete psychological capacities. Autonomy must be understood as the ongoing ability to notice influence, to pause and reflect, to resist momentary impulses, and to revise decisions over time. When these capacities are eroded, formal consent loses its ethical force, even if it remains legally valid (Wu and Yin, 2019).

Psychology therefore plays a central role in redefining the boundaries of acceptable persuasion. It provides the conceptual tools needed to distinguish influence that supports agency from influence that undermines it. Ethical persuasion is not defined by effectiveness or user satisfaction, but by its compatibility with cognitive sovereignty—the individual's capacity to govern their own attention, preferences, and decisions.

In this sense, reclaiming autonomy is not a call to eliminate persuasion, but to redesign its limits. Persuasive technologies can coexist with human freedom only if they are constrained by psychological principles that preserve awareness, self-regulation, and dignity. Without such constraints, intelligent persuasion risks normalizing forms of influence that quietly displace human agency rather than supporting it.

## Conclusion

It is a major challenge that AI-powered consumer nudging has transformed the nature of persuasion from transparent influence to algorithmic manipulation, raising urgent ethical and psychological concerns. Restoring control to users is essential and must be treated as a priority, not a choice. If we do not watch carefully, algorithmic persuasion can slowly harm people's dignity and ability to think carefully, which are needed for a good life. By including psychological knowledge in the main parts of AI design, and by supporting systems that improve rather than reduce freedom, we can find a better and more humane way forward.
